# A cross-sectional study to assess job strain of emergency healthcare workers by Karasek questionnaire: The SEEK study

**DOI:** 10.3389/fpsyt.2022.1043110

**Published:** 2023-01-05

**Authors:** Jean-Baptiste Bouillon-Minois, Marion Trousselard, Aurélien Mulliez, Oluwaseun John Adeyemi, Jeannot Schmidt, David Thivel, Ukadike Chris Ugbolue, Marjolaine Borel, Farès Moustafa, Guillaume T. Vallet, Maëlys Clinchamps, Marek Zak, Céline Occelli, Frédéric Dutheil

**Affiliations:** ^1^Université Clermont Auvergne, CNRS, LaPSCo, Physiological and Psychosocial Stress, CHU Clermont-Ferrand, Emergency Department, Clermont-Ferrand, France; ^2^French Armed Forces Biomedical Research Institute, Brétigny-sur-Orge, France; ^3^APEMAC/EPSAM, Metz, France; ^4^CHU Clermont-Ferrand, Clinical Research and Innovation Direction, Clermont-Ferrand, France; ^5^Ronald O. Perelman Department of Emergency Medicine, New York University Grossman School of Medicine, New York, NY, United States; ^6^Université Clermont Auvergne, Laboratory AME2P, Research Center in Human Nutrition, Aubière, France; ^7^Institute for Clinical Exercise and Health Science, University of the West of Scotland, Glasgow, United Kingdom; ^8^CHU Clermont–Ferrand, Emergency Medicine, Clermont-Ferrand, France; ^9^Unité de Nutrition Humaine (UNH), Institut National de la Recherche Agronomique (INRA), Université Clermont Auvergne, Clermont-Ferrand, France; ^10^Département de Psychologie, Université du Québec à Trois-Rivières, Trois-Rivières, QC, Canada; ^11^CNRS, LaPSCo, Physiological and Psychosocial Stress, Université Clermont Auvergne, Clermont-Ferrand, France; ^12^CHU Clermont-Ferrand, Occupational and Environmental Medicine, Clermont-Ferrand, France; ^13^Collegium Medicum, Institute of Health Sciences, Jan Kochanowski University of Kielce, Kielce, Poland; ^14^Department of Emergency, University Hospital, Nice, France

**Keywords:** emergency healthcare workers, burnout, public health, mental health, stress, emergency medicine

## Abstract

**Background:**

Emergency healthcare workers (eHCWs) are particularly at risk of stress, but data using the gold standard questionnaire of Karasek are scarce. We assessed the level of stress of eHCWs and aimed to compare it with the general population.

**Methods:**

This is a cross-sectional nationwide study in French Emergency Departments (EDs), using the job-content questionnaire of Karasek, compared with the 25,000 answers in the French general population (controls from the SUMER study). The descriptions of job demand, job control, and social support were described as well as the prevalence of job strain and isostrain. Putative factors were searched using mixed-method analysis.

**Results:**

A total of 166 eHCWs (37.9 ± 10.5 years old, 42% men) from five French EDs were included: 53 emergency physicians and 104 emergency paramedics, compared to 25,000 workers with other occupations. Job demand was highest for physicians (28.3 ± 3.3) and paramedics (25.9 ± 3.8), compared to controls (36.0 ± 7.2; *p* < 0.001). Job control was the lowest for physicians (61.2 ± 5.8) and paramedics (59.1 ± 6.8), compared to controls (70.4 ± 11.7; *p* < 0.001). Mean social support did not differ between groups (23.6 ± 3.4 for physicians, 22.6 ± 2.9 for paramedics, and 23.7 ± 3.6 for controls). The prevalence of job strain was massively higher for physicians (95.8%) and paramedics (84.8%), compared to controls (23.9%; *p* < 0.001), as well as for isostrain (45.1% for physicians, 56.8% for paramedics, and 14.3% for controls, *p* < 0.001). We did not find any significant impact of sociodemographic characteristics on job control, job demand, or social support.

**Conclusion:**

Emergency healthcare workers have a dramatic rate of job strain, necessitating urgent promotion of policy to take care of them.

## 1. Introduction

Stress at work is a main public health concern. In the medical field, half of the physicians are considered highly stressed (1). This is especially true in emergency departments (EDs) where healthcare workers (HCWs) have a complex interaction between stress due to shift work, fatigue, lack of sleep (2), poor food intake (3), cardiac strain (4), and life-threatening emergencies in a context of overcrowding (5, 6). Currently, the best scale to assess stress levels at work is the job-demand-control model (JDC), a self-reported psychological questionnaire, created and validated by Karasek in 1981 (7, 8). The JDC model recognizes the importance of daily environmental stressors on long-term experience of stress (9). It defines job demand and job control as the two broad work-related characteristics present in the environment of most occupations that could be stressful. Job demand refers to the psychological needs imposed by daily working activities, i.e., mental workload, organizational, and time constraints. Job control refers to the latitude of decision and is composed of two components, namely, skill discretion and decision authority. Each worker can perceive both job demand and job control at different levels. Karasek defined the combination of high job demands and low job control as “job strain,” the most aversive combination, at a risk of low wellbeing (10), burnout (11), and ill-health (12). On the other side, low job demand and high job control result in “low strain,” but this situation is rare. Since the 1980s, another dimension has been included in the JDC model, the “worksite social support”. Indeed, support from colleagues and/or from the hierarchy seems to act as a buffer against complex combinations of job control and demands (13). Isostrain is defined as job strain with low social support at work (14). The Job Content Questionnaire (JCQ) (15) derived from Karasek’s model has been developed and validated in several languages. The Karasek job strain model has been assessed among 25,000 workers in the French SUMER study and classified as the main types of occupations (14, 16). However, few or no data regarding emergency HCW (eHCW) scores in the Karasek questionnaire are available, even though burnout exposure is a well-known problem among them (17, 18). It seems that none of the sociodemographic characteristics [sex, age, body mass index (BMI), marital status, and physical activity] is a protective factor against job strain (19). However, in the case of association with “family strain” – family-related stress and familial conflict – women have more depression than men (20). Because eHCWs have a lot of risk factors that increase stress at work, we hypothesized a higher level among this population.

The main objective of this study was to assess stress levels using the Karasek JCD model among eHCWs and to compare them with the general population. The secondary objectives were to compare physicians and other providers (defined as paramedical) and to find the impact of sociodemographic characteristics on stress levels.

## 2. Materials and methods

### 2.1. Study design

**FIGURE 1:**
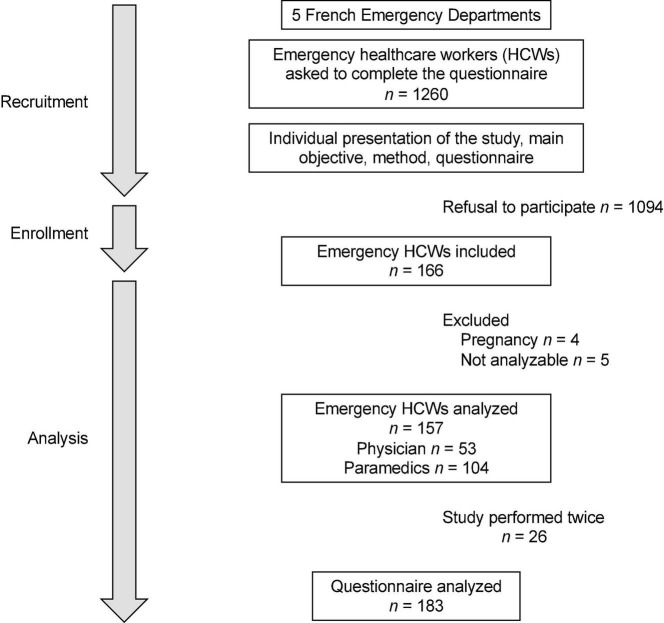
Study design. Among the five French EDs, we were able to recruit 192 emergency healthcare workers (eHCWs). Nine were excluded because of pregnancy or no data completion. A total of 183 Karasek surveys were analyzed from 157 eHCWs.

We performed an observational nationwide cross-sectional study. Volunteers of eHCWs were recruited from the French EDs. The study design is described in Figure 1. During the recruitment phase, all eHCWs individually receive an information letter attached by email. Acceptance or refusal was given by mail to avoid any subordination effect between the experimenter and the recruiter. If they agreed, they would have 8 days to sign the consent form to participate in the study. For privacy purposes, the question about their medical history was not asked by email. Instead, a list of non-inclusion criteria was given in the information letter. Exclusion criteria were refusal of participation, psychopathology with depression or anxiety, taking any drugs that modulate inflammation or hormone levels, and pregnancy. Participants were asked to complete the questionnaire between 6.30 and 9.00 a.m. We also used data from the SUMER study to compare the results from eHCWs to other occupations from the general population (14, 16).

### 2.2. Primary outcome

The primary outcome is the participants’ score on the French-validated JCQ questionnaire in the three dimensions, namely, psychological demands, decision latitude, and social support (21). Job demand and latitude decision-making were assessed by the 26 items of the JCQ (nine for both decision latitude and psychological demand and eight for social support). The subject was asked to respond using a 4-level Likert-type scale for each item, ranging from 1 (strongly disagree) to 4 (strongly agree). The decision latitude was calculated using the following formula: 4* Q4 +  4* (5 − Q6) +  4* Q8 +  2* (5 − Q2) +  2* Q5 +  2* Q7 + 2* Q1 +  2* Q3 +  2* Q9. A score below 71 reflects low decision latitude. The psychological demand was calculated using the following formula: Q10 + Q11 + Q12 + (5 − Q13) + Q14 + Q15 + Q16 + Q17 + Q18. A score below 20 reflects a low psychological demand. Finally, the social support was calculated using the following formula: Q19 + Q20 + Q21 + Q22 + Q23 + Q24 + Q25 + Q26. A score below 24 reflects low social support (15). In a previous study performed by our team, Cronbach’s alphas for job demands, job control, and social support were 0.58, 0.99, and 0.99, respectively (22). By combining autonomy and demand, four broad categories are defined: (1) Relaxed work: low demand and high autonomy; (2) Passive work: low demand and low autonomy; (3) Active work: high demand and high autonomy; and (4) Stressed, tense work: high demand and low autonomy. Furthermore, job strain was defined as a demand score higher than 21 and a control score less than 70 (16). A social support level < 24 denotes isostrain.

### 2.3. Secondary outcomes

We collected sociodemographic characteristics such as age, sex, weight, height, kids at home, and marital status. Physical activity (in hours per week), sleep quantity (in hours), and quality using a visual analog scale from 0 (bad quality) to 100 (good quality) of every HCWs were asked. We also collected information about the ED and the type of hospital (university or not), as well as. Finally, seniority (within the ED and as an emergency occupation). We defined the class as “physician” if the responder was attending, fellow, or resident. We defined the class as “paramedics” if the responder was a nurse, caregiver, cleaner, or administrative job. We studied the impact on job demand, job control, social support, job strain, and isostrain for each following criteria: sociodemographic characteristics, seniority, tobacco, coffee or tea, job, and night vs. day.

### 2.4. Statistics

No sample size was computed for this purpose as this is an ancillary study of the SEEK protocol [NCT02401607 (23)], in which 192 subjects were included. We reused those data in order to assess Karasek’s scores among eHCWs. In this study, 157 subjects were included. The study sample was described by frequency and percentage for categorical data and by mean ± standard deviation for continuous data when the distribution was normal, or otherwise by median and interquartile range. The normality assumption was assessed graphically using the Shapiro–Wilk test. A comparison between groups (physicians vs. paramedics), considering the subject as a statistical unit, was performed using Student’s *t*-test (or Mann–Whitney *U* test when data are not normal) for continuous data and using the χ^2^ test (or Fisher’s exact test when appropriate) for categorical data. Analyses considering the measures (job control, job demand, and social support) as a statistical unit, considering repeated measures for some subjects, were performed using linear mixed models, with the subject as a random effect, first in a univariate approach in order to identify characteristics associated with the three scores, and then in a multivariable linear mixed model, adjusting for factors statistically highlighted in univariate analysis or clinically relevant. Results are shown as regression coefficients and their 95% confidence intervals. For job strain and isostrain outcomes, the process was similar except for using a logistic mixed model, and results were presented as odds ratios and their 95% confidence intervals. Statistics were performed using Stata [StataCorp; Stata statistical software: Release 16. College Station, TX, USA: StataCorp LLC]. This study was performed in accordance with the Declaration of Helsinki and each participant signed a consent form. A French ethics committee (Comité de Protection de Personnes Sud-Est I, CHU Saint-Etienne) approved this study protocol on 3 November 2014, with reference DC-2014-2151. This protocol was registered in Clinical Trials under the identification NCT02401607.

## 3. Results

### 3.1. Characteristics of the population

We recruited 166 eHCWs from five French EDs, before the COVID-19 pandemic. Nine eHCWs were excluded because of pregnancy (*n* = 4) and incomplete data (*n* = 5; Figure 1). A total of 157 eHCWs (53 physicians and 104 paramedics) were analyzed and compared with the 25,000 answers in the French general population (controls from the SUMER study) (14, 16). eHCWs had a mean age of 37.5 ± 10.5 years old: 35.5 ± 10.5 among physicians and 38.5 ± 10.3 among paramedics. There were 66 (42%) men: 27 (50.9%) men among physicians and 39 (37.5%) men among paramedics. BMI was 23.4 ± 3.3 kg/m^2^. Physicians had a median seniority of 3.5 [P25–P75: 1.5–10] years on the job and 1.25 [0.5–5] in the ED, while paramedics had 10 [4–17] years on the job and 4 [1–11] years in the ED. They drank 3 [2–4.5] cups of coffee or tea per day and performed 3 [1–4] h of physical activity per week. Table 1 describes all sociodemographic characteristics. Twenty-six of them performed the study twice, so we were able to analyze 183 surveys.

**TABLE 1:** Characteristics of the population.

	Total *n* = 157	Physician *n* = 53	Paramedics *n* = 104	*p*-value
Age (years), mean ± SD	37.5 ± 10.5	35.5 ± 10.5	38.5 ± 10.3	**0.046**
Men [*n* (%)]	66 (42.0)	27 (50.9)	39 (37.5)	0.11
Physical activity (h/week), median [IQR]	3 [2–4]	3 [2–5.5]	3 [2–4]	0.71
**Body mass index (kg/m^2^), mean ± SD**	23.4 ± 3.5	23.3 ± 2.9	23.5 ± 3.8	0.94
Underweight [*n* (%)]	8 (5.2)	2 (3.9)	6 (5.8)	
Normal weight [*n* (%)]	105 (67.7)	37 (71.2)	68 (66.0)	
Overweight [*n* (%)]	37 (23.9)	13 (25.0)	24 (23.3)	
Obesity class 1 [*n* (%)]	4 (2.6)	0	4 (3.9)	
Obesity class 2 [*n* (%)]	1 (0.7)	0	1 (1.0)	
**Seniority (years)**				
Job, median [IQR]	6 [3–17]	3.5 [1.5–10]	10 [4–17]	**<0.001**
Department, median [IQR]	3 [1–10]	1.25 [0.5–5]	4 [1–11]	**0.003**
**Type of hospital**				0.19
General	74 (47.8)	20 (38.5)	54 (52.5)	
University	81 (52.3)	32 (61.5)	49 (47.6)	
Tea-coffee (cup/shift), median [IQR]	3 [2–4.5]	4 [3–5]	3 [2–4.3]	0.14
Smoker [*n* (%)]	52 (33.1)	15 (28.3)	37 (35.6)	0.36
Sleep at home quantity (h), mean ± SD	7.3 ± 1.1	7.2 ± 0.9	7.3 ± 1.2	0.99
Sleep at home quality (VAS), mean ± SD	68.5 ± 20.3	73.0 ± 19.5	66.3 ± 20.4	0.065
**Family situation**				0.28
Single-divorced [*n* (%)]	50 (32.1)	20 (37.7)	30 (29.1)	
Married-engaged [*n* (%)]	106 (67.9)	33 (62.3)	73 (70.9)	
Kids at home [*n* (%)]	50 (39.7)	13 (30.2)	37 (44.6)	0.12

157 eHCWs were included. Results are expressed in mean ± standard deviation or number (percentage) or median [first quartile-third quartile], SD, standard deviation; kg/m^2^, kilogram per square meters; VAS, visual analog scale from 0 (worst quality ever) to 100 (best quality ever). Results are significant if *p* < 0.05. Bold values means significant results.

### 3.2. The main objective of the job content questionnaire of Karasek

**FIGURE 2:**
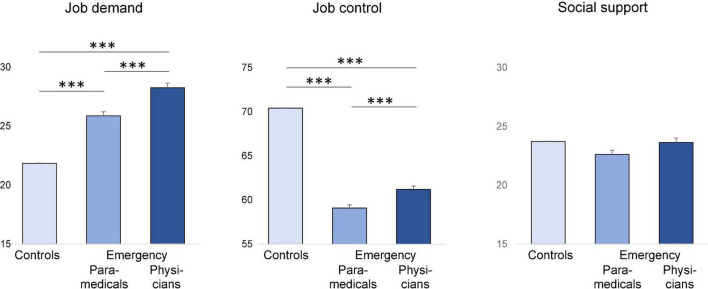
Comparison of job demand, job control, and social between controls (SUMER), paramedics, and emergency physicians from emergency departments. ^***^p < 0.001. Results are significant if *p* < 0.05.

**FIGURE 3:**
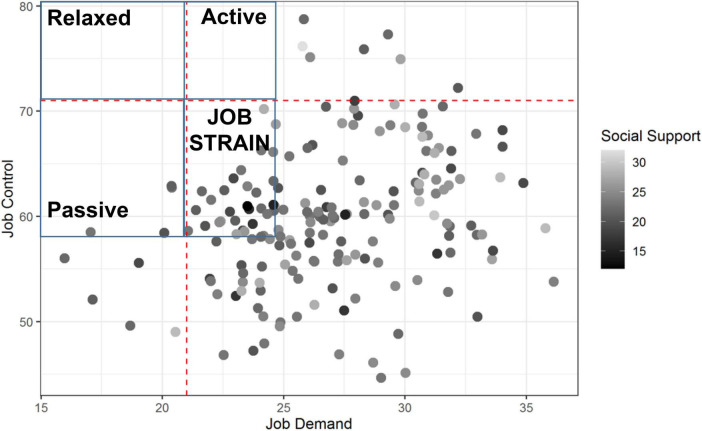
All occupations from SUMER studies (>100,000 French participants) were within the four colored quadrants. Each dot represents an emergency healthcare worker from our study. Darker dots represent lower social support. Study of the validity of a job-exposure matrix for psychosocial work factors: results from the national French SUMER survey. Int Arch Occup Environ Health 82: 87–97; 15 (24).

**FIGURE 4:**
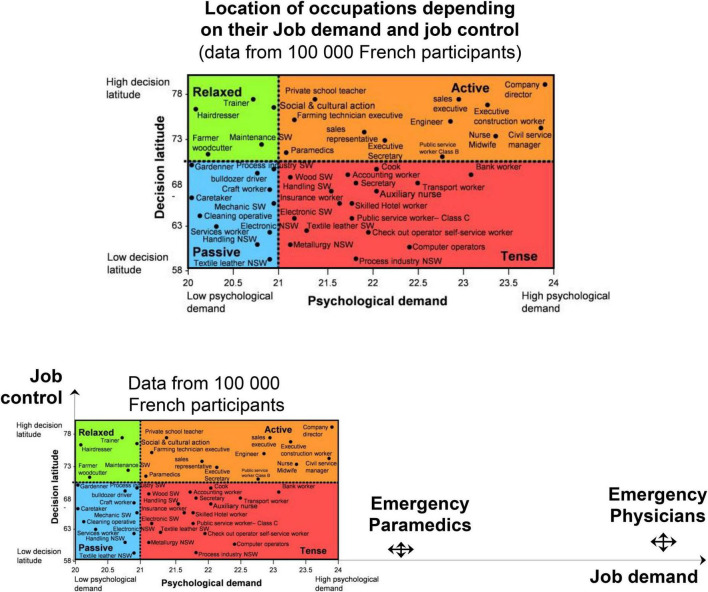
Representation of emergency physicians and paramedics compared to other jobs. Results of other jobs are from the study of the validity of a job-exposure matrix for psychosocial work factors: results from the national French SUMER survey. Int Arch Occup Environ Health 82: 87–97; 15 (24).

**FIGURE 5:**
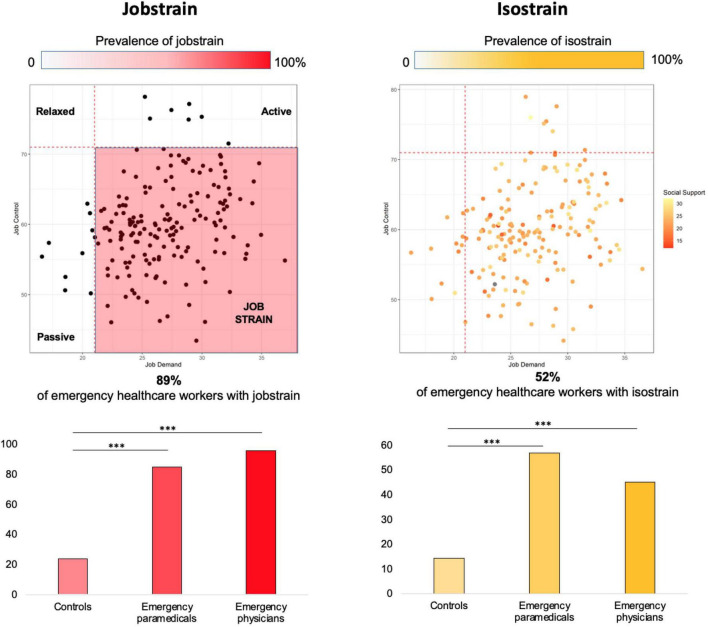
Prevalence of job strain and isostrain among our population of emergency healthcare workers. ^***^p < 0.001. Results are significant if *p* < 0.05. Patients are considered in job strain if job demand is >21 and job control is <70. Patients are considered isostrain if they have job strain and social support < 24.

Job demand was highest for physicians (28.3 ± 3.3) and paramedics (25.9 ± 3.8), compared to controls (36.0 ± 7.2; *p* < 0.001). Job control was the lowest for physicians (61.2 ± 5.8) and paramedics (59.1 ± 6.8), compared to controls (70.4 ± 11.7; *p* < 0.001). Mean social support did not differ between groups (23.6 ± 3.4 for physicians, 22.6 ± 2.9 for paramedics, and 23.7 ± 3.6 for controls) (Figure 2). The prevalence of job strain was massively higher for physicians (95.8%) and paramedics (84.8%), compared to controls (23.9%; *p* < 0.001), as well as for isostrain (45.1% for physicians, 56.8% for paramedics, and 14.3% for controls, *p* < 0.001; Figures 3–5).

### 3.3. Impact of sociodemographic characteristics

**FIGURE 6:**
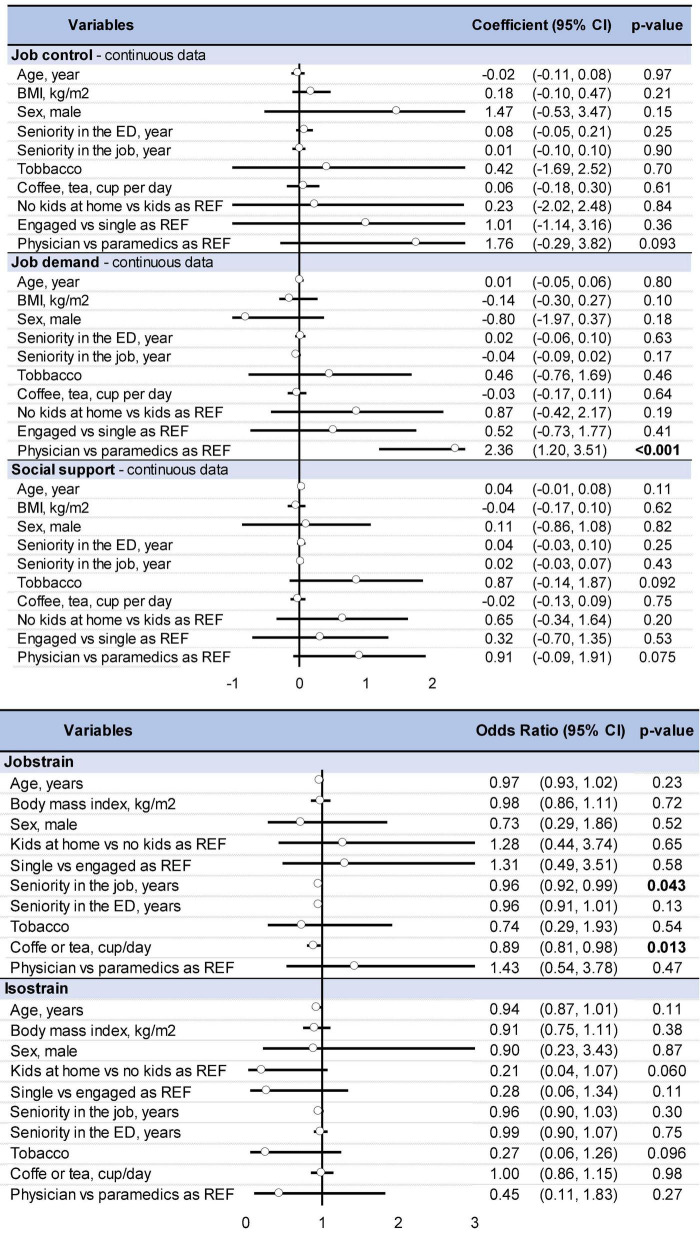
Impact of sociodemographic characteristics on quantitative data (job control, job demand, and social support) and qualitative data (job strain and isostrain). Results about quantitative data are a coefficient and those about qualitative data are in odds ratio. Bold values means significant results.

Mixed models showed that physicians have a higher level of job control, job demand, and social support (Figure 5). They were also more job strain compared to paramedic HCWs. Drinking coffee and job experience decrease job strain (OR = 0.89; 0.81–0.98 and 0.96, 0.92–0.99, respectively). Although not significant, kids seem to be a protective factor of isostrain (OR = 0.21, 0.04–1.07). We did not find any significant impact of age, BMI, sex, tobacco, coffee, seniority on job control, job demand, or social support (Figure 6).

## 4. Discussion

The main objective of this study was to assess job strain among eHCWs. Although we were confident to find a high rate of job strain, our findings were due to an association of high job demand with low job control. Fortunately, the relatively high social support between eHCWs seems to counterbalance their high job strain.

### 4.1. Karasek’s model

A recent study proposed that the use of visual analog scales is more effective than a long survey (25). According to the SUMER study that assessed Karasek’s survey among 25,000 French employees, the French version of the survey has good validity, especially for job control (16). However, no eHCW was included in this study. High psychological demand is defined as job demand higher than 21. Therefore, eHCWs are in a situation that could qualify as “non-standard” compared to the French population. Indeed, the mean job demand was 20.99 when we found 26.3. Furthermore, they have low job control, i.e., less than 70. The French population had a 70.32 level of job control while our population had a 59.9 level. Finally, the score obtained for the social support item is almost identical between the SUMER study and our population (23.34 vs. 23.0). Regarding job strain, we found an extreme score of 89.1 vs. 23.9% in the SUMER study (16). Considering that job strain is a risk factor for musculoskeletal pain (26), coronary heart disease (27, 28), type 2 diabetes mellitus (29), cancer (30), depression (31), burnout (32), and mortality (33), our results are alarming but some explanations could be done. Indeed, this feeling of a tense situation at work is probably linked to the number of tense relations with the public, overcrowding, lack of availability of beds, stressful event, patient’s stress, manual handling, or even time constraints (4–6). Another explanation is stress contagion. Indeed, stress, like all other emotions, is contagious (34). This contagion can occur between caregivers and also between patients and HCWs (35). Considering that consulting in an ED is a stressful event, it could be relevant to adjust HCWs’ stress level with the patient’s level. Furthermore, it seems that physicians can absorb joy and anger from their colleagues and nurses from leaders, colleagues, and patients. Joy and anger-absorbed were related to the physician’s exhaustion and cynicism (36).

### 4.2. All emergency HCWs, but more especially physicians

We compared EPs and other eHCWs and showed that physicians have a higher score in job demand, job control, and social support. Furthermore, we found a higher rate of job strain and isostrain. Previous studies have shown that nurses have high cognitive and sensory demands with a low degree of freedom at work, a lack of autonomy, numerous duties, great meaning and commitment to work, less social support, and a lack of feedback at work (37–39). Although the concept of shared medical decision-making exists for decades, it is poorly used in daily practice between nurses and physicians (40). Indeed, this requires decision-making on a multi-daily basis, leading, in the very short term, to a vital stake in the state of health of patients, and this, in a context of stress, is reinforced by the increasingly frequent recourse to justice in the event of a medical error (41). The physician, compared to other eHCWs, retains the central role of decision-maker, which can partially explain the obtaining of a higher score vs. other eHCWs. Furthermore, nurses share the belief that the physician should decide and the patient should rely on his knowledge rather than his own (42). Finally, some studies found a direct link between physician burnout (in which job strain is one of the main risk factors) and adverse patient outcomes (43). This indicates that it increases the stress of the physician, which increases the job strain and the vicious circle begins. Although we found a higher rate of isostrain, we also found a higher level of social support among physicians compared to other eHCWs. HCWs and, especially, physicians have a long history of support between them, sometimes considered confraternity or brotherhood (44, 45). Social support can have an important impact on care because it partially mediates the relationship between physician burnout and behavior-based professionalism (46). Furthermore, it is a target to improve quality of life and decrease burnout (47, 48).

### 4.3. Limitations

Our study has some limitations. Questionnaires were filled out by volunteers and a selection bias may have occurred, however, the large sample size may limit this bias, as well as the multicentric recruitment (49). As for all questionnaires, self-reported information may overestimate or underestimate the sensations of job demand, job control, and social support. This study was performed before the COVID-19 pandemic (5, 6), limiting heterogeneity between measurements over time. However, repetition in the near future of the collection of data may promote a longitudinal follow-up of eHCWs. Although the JDC model of Karasek is the gold standard to assess psychosocial risks at work (8), its length makes it difficult to use routinely in daily clinical practice by occupational practitioners and we recently proposed validation of visual analog scales of job demand and job control (25). Unfortunately, we failed to demonstrate the putative influence of some sociodemographic characteristics on job strain or isostrain, except for the role of seniority within the job that decreased the risk of job strain, in accordance with the literature (50). We also found that coffee consumption is linked with a lower feeling of job strain, which could be explained by the anti-stress properties of caffeine (51).

## 5. Conclusion

Emergency healthcare workers work under stressful condition that induces a dramatic rate of job strain. It could possibly be explained by several factors such as overcrowding, a lack of time, and shift work. We must create policies to generate a safer place to take care of our workers.

## Data availability statement

The raw data supporting the conclusions of this article will be made available by the authors, without undue reservation.

## Ethics statement

The studies involving human participants were reviewed and approved by the Comité de Protection de Personnes Sud-Est I, CHU Saint-Etienne on 3 November 2014, with reference DC-2014-2151. This protocol was registered in Clinical Trials under the identification NCT02401607. The patients/participants provided their written informed consent to participate in this study.

## Author contributions

J-BB-M and FD: conceptualization. J-BB-M, MT, JS, and FD: methodology. AM: formal analysis. MB, FM, FD, and JS: investigation. FD: data curation. J-BB-M: writing — original draft preparation. DT, MT, AM, OA, UU, MB, FM, MC, GV, MZ, and CO: writing — review and editing. J-BB-M, AM, and FD: visualization. All authors have read and agreed to the published version of the manuscript.
